# Genetic Regulation of Cytokine Response in Patients with Acute Community-Acquired Pneumonia

**DOI:** 10.3390/genes13010111

**Published:** 2022-01-06

**Authors:** Andreas Kühnapfel, Katrin Horn, Ulrike Klotz, Michael Kiehntopf, Maciej Rosolowski, Markus Loeffler, Peter Ahnert, Norbert Suttorp, Martin Witzenrath, Markus Scholz

**Affiliations:** 1 Institute for Medical Informatics, Statistics and Epidemiology, Medical Faculty, Leipzig University, 04103 Leipzig, Germany; katrin.horn@imise.uni-leipzig.de (K.H.); ulrike.klotz@imise.uni-leipzig.de (U.K.); maciej.rosolowski@imise.uni-leipzig.de (M.R.); markus.loeffler@imise.uni-leipzig.de (M.L.); peter.ahnert@imise.uni-leipzig.de (P.A.); markus.scholz@imise.uni-leipzig.de (M.S.); 2 Institute for Clinical Chemistry and Laboratory Diagnostics, Jena University Hospital, 07740 Jena, Germany; michael.kiehntopf@med.uni-jena.de; 3 Division of Infectiology and Pneumonology, Medical Department, Charité—Berlin University Medicine, 13353 Berlin, Germany; norbert.suttorp@charite.de (N.S.); martin.witzenrath@charite.de (M.W.)

**Keywords:** cytokine, genome-wide association study, conditional and joint analysis, colocalisation analysis, MetaXcan analysis

## Abstract

Background: Community-acquired pneumonia (CAP) is an acute disease condition with a high risk of rapid deteriorations. We analysed the influence of genetics on cytokine regulation to obtain a better understanding of patient’s heterogeneity. Methods: For up to *N* = 389 genotyped participants of the PROGRESS study of hospitalised CAP patients, we performed a genome-wide association study of ten cytokines IL-1β, IL-6, IL-8, IL-10, IL-12, MCP-1 (MCAF), MIP-1α (CCL3), VEGF, VCAM-1, and ICAM-1. Consecutive secondary analyses were performed to identify independent hits and corresponding causal variants. Results: 102 SNPs from 14 loci showed genome-wide significant associations with five of the cytokines. The most interesting associations were found at 6p21.1 for VEGF (*p* = 1.58 × 10^−20^), at 17q21.32 (*p* = 1.51 × 10^−9^) and at 10p12.1 (*p* = 2.76 × 10^−9^) for IL-1β, at 10p13 for MIP-1α (CCL3) (*p* = 2.28 × 10^−9^), and at 9q34.12 for IL-10 (*p* = 4.52 × 10^−8^). Functionally plausible genes could be assigned to the majority of loci including genes involved in cytokine secretion, granulocyte function, and cilial kinetics. Conclusion: This is the first context-specific genetic association study of blood cytokine concentrations in CAP patients revealing numerous biologically plausible candidate genes. Two of the loci were also associated with atherosclerosis with probable common or consecutive pathomechanisms.

## 1. Introduction

Community-acquired pneumonia (CAP) is an acute inflammatory condition of the lung acquired outside of the health care system. It affects people of all ages. The disease is characterised by a risk of rapid deterioration with high mortality, which is difficult to predict. Thus, hospitalisation and narrow surveillance of patients is often required [1].

CAP has high inter-individual heterogeneity due to the complex regulation of the immune system comprising highly non-linear dynamics [2]. Cytokines released during inflammatory response were shown predictive for treatment failure and mortality [3,4]. We showed in the past that cytokine dynamics are causally related to relevant clinical outcome parameters [5].

Genetic determinants of immune response are poorly investigated due to the fact that cross-sectional data of cytokines in population-based cohorts are less informative for acute conditions while patients with acute disease are particularly difficult to collect. Genome-wide association analyses comprised the impact of MCP-1 on the risk of stroke [6], the pharmacogenomics of rheumatoid arthritis treatment using anti-TNF therapy [7], the causal role of cytokines in immune-related and chronic diseases [8], the comorbidity of schizophrenia with tuberculosis identifying common cytokines involved [9], and pleiotropic effects on cytokines [10].

We established the PROGRESS study collecting data of about three thousand hospitalised CAP patients at baseline and for four to five consecutive days [11]. Using this resource, we aim to unravel genetic determinants of cytokine response of CAP patients. We performed a genome-wide association study, and consecutively, secondary analyses to identify novel loci of context-specific cytokine response and to corroborate other candidate loci.

## 2. Materials and Methods

### 2.1. Study Sample

Participants were recruited within the framework of the PROGRESS study (clinicaltrials.gov identifier: NCT02782013). PROGRESS is a multi-center clinical observational study of hospitalised patients with CAP with ongoing recruitment since August 2009. Cytokine levels used in our analyses were measured in patients recruited from September 2009 until January 2013. Details of the study design and inclusion/exclusion criteria can be found in [11]. In brief, patients were included if they were 18 or more years old and had a working diagnosis of pneumonia. Patients were excluded if they stayed in hospital during the previous 28 days or if they were hospitalised for more than 48 h before enrollment to avoid recruitment of patients with nosocomial infections, i.e. hospital-acquired pneumonia. Patients with HIV infection, AIDS, or immunosuppressive treatments within the past six months, pregnancy, or other lung diseases were also excluded. Data collection includes daily measurements of parameters of disease severity such as the Sequential Organ Failure Assessment (SOFA) score and laboratory parameters [12]. Additionally, patients were also characterised for a number of molecular layers including genetics, transcriptomics, and proteomics.

### 2.2. Cytokine Measurement and Analysis

We used data from cytokine measurements described in [5]. Briefly, the cytokines IL-1β, IL-6, IL-8, IL-10, IL-12, MCP-1 (MCAF), MIP-1α (CCL3), VEGF, VCAM-1, and ICAM-1 were determined in serum by a LUMINEX based multiplex Bead Array System (Luminex 200, Luminex, DiaSorin, Austin, TX, USA). Only measurements of the day of admission of each patient were considered, resulting in a sample size of *N* = 400. Baseline statistics of study participants are provided in Table 1.

Data analyses was carried out cytokine-wise. Values below the limit of detection (LOD) were set to missing. Values not under LOD were transformed by natural logarithm. Outlier detection was performed by exclusion of values more than four inter-quartile ranges above the third quartile respectively below the first quartile. This removed 1 sample for VEGF and 2 samples for VCAM-1 and ICAM-1 each. We adjusted for batch effects using Empirical Bayes method as implemented in the package ComBat [13] of the statistical software suite “R”.

### 2.3. Genotyping, Quality Control, and Imputation

Genotypes were measured by the CAP2 array which is a customised SNP microarray based on the Axiom platform (Affymetrix, Santa Clara, CA, USA). The CAP2 array comprises the standard genome-wide content of the Axiom Genome-Wide CEU 1 Array Plate and about 70,000 custom SNPs resulting in a total of 659,675 SNPs. Custom SNPs were selected from the literature and eQTL, GWAS, and functionally relevant variant data bases.

A total of *N* = 2277 samples were genotyped. Genotype calling was performed with Affymetrix Power Tools (APT) software (version 2.10.2.2) with standard settings. Sample and SNP quality control (QC) were performed using the software R (version 3.5.2). During sample QC, samples were excluded if one or more of the following filtering criteria were fulfilled: Dish QC (signal to noise ratio) < 0.82, call rate < 0.97, differences between submitted and genotyped sex, and implausible relatedness. Genetic heterogeneity was assessed by principal component analysis (PCA). Outliers were excluded if six standard deviations away from the mean. For SNP QC, we excluded SNPs with a call rate < 0.97, Fisher’s Linear Discriminant (FLD) < 3.6, Heterozygous Cluster Strength Offset (HetSO) < −0.1, Homozygote Ratio Offset (HomRO) < −0.9 (for three clusters), violation of Hardy–Weinberg equilibrium (HWE) (*p* ≤ 1 × 10^−6^ in exact test), and plate association (*p* ≤ 1 × 10^−7^ in χ^2^-test). After QC, a total of *N* = 2174 samples and M = 600,567 SNPs were available.

IMPUTE2 software (version 2.3.2) was used for imputation along with the 1000 Genomes Project reference data base (phase 3, version 5) [14]. Imputation increased the number of SNPs to M = 85,064,535. We only considered associations for SNPs with minor allele frequency (MAF) ≥ 0.01 and imputation info score ≥ 0.8 resulting in M = 9,140,487 markers.

### 2.4. Genome-Wide Association Study

Combined genotype and cytokine data were available for a minimum of *N* = 361 and a maximum of *N* = 389 samples depending on the cytokine (Table S1). Associations between genotypes and cytokines were analysed by an additive linear regression model using software PLINK (v2.00a2LM AVX2 Intel (28 Oct 2018)). X-chromosomal markers were analysed assuming total X-inactivation (i.e., male genotypes are coded as 0/2 while female genotypes are coded as 0/1/2). *p*-values less than or equal to 5 × 10^−8^ were considered genome-wide significant. Suggestive SNPs are defined by *p*-values larger than 5 × 10^−8^ but less than or equal to 1 × 10^−6^. Top-hits were priority pruned by applying a linkage disequilibrium (LD) cut-off of *r*^2^ ≥ 0.3 using the 1000 Genomes Project reference data base (phase 3, version 5) [14] as LD reference.

SNPs were annotated by nearby genes (nearest three genes within ±250 kilo base pairs, kb) using Ensembl [15], with other trait associations by LD-based lookup (*r*^2^ ≥ 0.3) in the GWAS Catalog [16], and with expression quantitative trait loci (eQTLs) by LD-based lookup (*r*^2^ ≥ 0.3) using the Genotype-Tissue Expression data base (GTEx) [17] and (updated) own data [18].

### 2.5. Conditional and Joint Analysis

To identify further independent hits per locus, we considered the best associated trait and performed conditional analyses by applying the tool GCTA (version 1.92.0beta3) [19]. First, we performed stepwise model selection (“CoJo-Slct”) to identify the independent variants per locus. As LD reference panel we used the complete set of genotypes (*N* = 2174). In case of multiple variants per locus, conditional effect estimates were calculated using “CoJo-Cond”.

### 2.6. Credible Set Analysis

After determination of the independent signals, we aimed at identifying the respective set of SNPs containing the causal variant with high certainty. For this purpose, we considered the set of SNPs within ±500 kb of the independent lead SNPs and their respective (conditional) effect estimates and standard errors [20,21]. We then calculated respective Approximate Bayes Factors (ABF) by applying the R-package “gtx”. The required prior distribution of the standard deviation was constructed empirically by the difference of the 97.5th and the 2.5th percentile of SNP effects of the respective locus divided by 2 × 1.96. In our data, this quantity ranged in between 0.1485 (locus 2p16.3) and 0.3074 (locus 18q21.2).

### 2.7. Colocalisation Analysis

We tested whether the independent loci coincide with loci of eQTLs of candidate genes in whole blood. EQTLs were retrieved from GTEx Analysis V8 (dbGaP Accession phs000424.v8.p2) [22]. Colocalisation analysis evaluates the posterior probability of five hypotheses:

**Hypothesis** **0** (**H0).** *No associations within locus.*

**Hypothesis** **1** (**H1).** *Associations with trait 1 (cytokine) only.*

**Hypothesis** **2** (**H2).** *Associations with trait 2 (gene expression) only.*

**Hypothesis** **3** (**H3).** *Association with both traits but different SNPs (no colocalisation).*

**Hypothesis** **4** (**H4).** *Association with both traits with the same SNP—evidence for colocalisation.*

We consider a minimum posterior probability of 0.75 as sufficient to support one of the hypotheses. Loci were again defined by a ±500 kb window around the respective lead SNPs.

### 2.8. MetaXcan Analysis

Summary statistics of all cytokines were used to discover correlations with respective genetically estimated gene expressions using the MetaXcan approach [23]. The gene expression prediction models were downloaded from PredictDB (GTEx Analysis V8). For analyses, we used all available 49 tissues from GTEx and performed hierarchical false discovery rate correction to adjust for multiple testing (levels: cytokines and tissues).

### 2.9. Lookup of Cytokine Coding Genes

We searched for associations in the genes coding for the cytokines analysed. This is performed by considering SNPs in the respective gene body with a ±500 kb margin around gene start and stop using Genome Reference Consortium Human Build 38. To account for multiple testing, we performed Benjamini–Hochberg procedure in a hierarchical manner.

## 3. Results

### 3.1. Genome-Wide Association Study and Secondary Analyses

In our GWAS analysis of ten cytokines, no signs of general inflation of test statistics were detected (λ in between 0.9934 to 1.0140, c.f. Table S2). We found 102 SNPs genome-wide significantly associating with at least one cytokine. Five of the ten cytokines were involved in genome-wide associations. SNPs could be assigned to 14 genomic loci. For all loci, there was only one independent variant according to conditional and joint analyses (CoJo-Slct). Colocalisation with blood eQTLs was found for only one locus.

**Figure 1:**
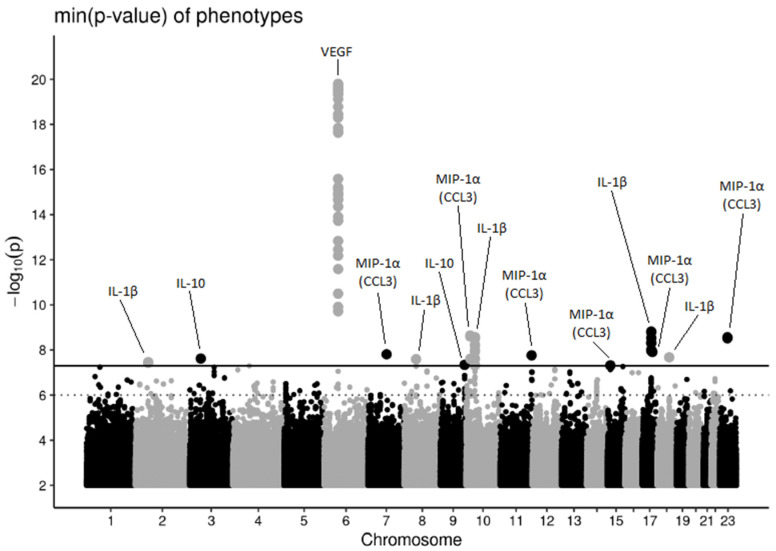
Manhattan plot showing association results of the ten considered cytokines. For each SNP the maximum negative log-*p*-value with respect to all cytokines is shown. The solid line corresponds to the genome-wide significance threshold (5 × 10^−8^). The dotted line indicates suggestive associations with a *p*-value less than or equal to 1 × 10^−6^ for one of the cytokines. Associations could be assigned to 14 distinct loci. Genome-wide significant hits are annotated by their best associated cytokine.

A visual overview of GWAS results is given by the Manhattan plot across all cytokines in Figure 1 and corresponding locus-wise statistics are provided in Table 2 (for a comprehensive overview of the 14 loci we provide Table S3). Regional association plots of all loci are provided as Figure S1.

**Figure 2:**
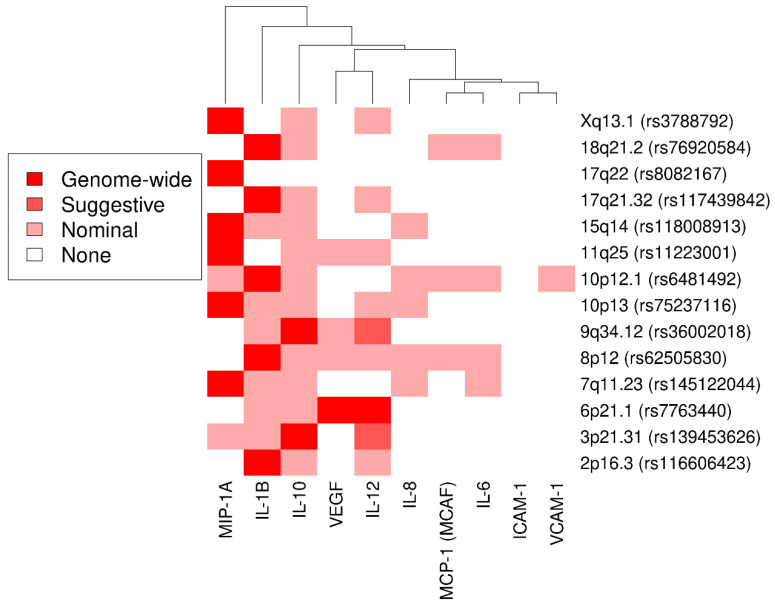
Cluster heatmap for associations between the lead SNPs of the 14 loci and the 10 cytokines of interest. Strength of association is indicated by color intensity. Cytokines are ordered by hierarchical clustering according to *p*-value similarity.

We further illustrated the strength of association between the 14 loci (lead SNP) and all considered cytokines in Figure 2. Noteworthy, only locus 6p21.1 showed genome-wide significant association with two cytokines. For loci 3p21.31 and 9q34.12, two additional cytokines were associated with suggestive significance. All loci except for 17q22 showed nominally significant co-associations for up to six cytokines. *p*-value–based hierarchical clustering showed grouping of cytokines across all 14 loci whereas each locus seemed to affect mainly one cytokine.

### 3.2. Known Associations

#### 3.2.1. Vascular Endothelial Growth Factor and Interleukin 12

The strongest association was found at 6p21.1 for VEGF (rs7763440, *p* = 1.58 × 10^−20^). The lead SNP also showed genome-wide significance with IL-12 (strongest association for rs4320361, *p* = 2.31 × 10^−9^, linkage disequilibrium with lead SNP: LD = 0.9976). Both effect directions (VEGF and IL-12) were negative for the lead SNP. The locus was already reported for associations with VEGF [24] and blood protein levels [25]. Further associations with multiple cancers [26] and ischemic stroke (especially large artery atherosclerosis, LAA) [27] were also reported. The lead SNP is near *C6orf223*, *MRPL14*, *TMEM63B*, and *VEGFA*, where the latter is the obvious candidate gene. Furthermore, for rs7763440, we could identify the following cis-eQTL genes: *CAPN11*, *HSP90AB1*, *MRPL14*, *RSPH9*, *SLC29A1*, and *SLC35B2*. Of note, *RSPH9* was reported to be associated with primary ciliary dyskinesia [28]. The 99% credible set for the independent lead SNP rs7763440 comprises 12 SNPs. Another associated tag-SNP rs7739450 at this locus (*p* = 1.92 × 10^−10^) showed a CADD score of 10.84. The lead SNP rs7763440 colocalises with an eQTL of *C6orf223* in whole blood (posterior probability PP = 93.7%). For VEGF and IL-12, significant association according to hierarchical false-discovery rate with *C6orf223* in Whole Blood could be detected by MetaXcan analysis. In both cases, three SNPs were involved in model analyses.

#### 3.2.2. Interleukin 1β

The second strongest association was rs117439842 at 17q21.32 with IL-1β (*p* = 1.51 × 10^−9^). The SNP is located in proximity to *SP6*, *SCRN2*, *LRRC46*, *MRPL10*, *OSBPL7*, and *SP2*. Associations with this locus were reported for this cytokine [29] but also for primary ciliary dyskinesia [30] and epilepsy (genetic generalized epilepsy, genetic absence epilepsy, juvenile myoclonic epilepsy) [31]. The 99% credible set for the independent lead SNP rs117439842 consists of 23 SNPs. MetaXcan analysis revealed significant associations according to hierarchical false-discovery rate with 13 genes in all tissues except Heart Atrial Appendage using a median number of 2 SNPs (IQR = 1–3, Range = 1–5) within model analyses (c.f. Table S4). *SCRN2*, *LRRC46*, and *SP2* are considered as plausible candidate genes.

### 3.3. Novel Associations

#### 3.3.1. Interleukin 1β

A strong association was detected at 10p12.1, again, with IL-1β (rs6481492, *p* = 2.76 × 10^−9^). The SNP rs6481492 is in *ARMC4* and in the near of *RPL36AP55*, *MPP7*, and *RN7SKP132*. Moreover, for the lead SNP, we could identify the cis-eQTL genes *ABI1*, *ARMC4*, *BAMBI*, *MASTL*, *RAB18*, and *WAC*. *ARMC4* was reported to be associated with primary ciliary dyskinesia [32] and vital capacity [33]. For rs6481492, the 99% credible set comprises 50 SNPs. The tag-SNP rs144080867 at this locus (*p* = 4.66 × 10^−8^) revealed a CADD score of 11.04. In conclusion, *ARMC4* is a plausible candidate gene.

Other associations for this cytokine could be found at loci 2p16.3 (rs116606423, *p* = 3.46 × 10^−^^8^), 8p12 (rs62505830, *p* = 2.55 × 10^−^^8^), and 18q21.2 (rs76920584, *p* = 2.11 × 10^−^^8^). However, for these three loci, we cannot suggest any obvious candidate genes.

#### 3.3.2. Interleukin 10

For IL-10, we found an association of rs36002018 at 9q34.12 (*p* = 4.52 × 10^−^^8^). The SNP is in *ABL1* and in proximity of *EXOSC2*, *PRDM12*, and *QRFP*. *ABL1* is a proto-oncogene that encodes a protein tyrosine kinase involved in a variety of cellular processes, including cell division, adhesion, differentiation, and response to oxidative stress [30]. The gene is further involved in chronic myeloid leukemia [30]. Thus, *ABL1* is a plausible candidate gene.

Another association was found at 3p21.31 for rs139453626 (*p* = 2.39 × 10^−^^8^). The SNP is in *SMARCC1*, which belongs to the neural progenitors-specific chromatin remodeling complex (npBAF complex) and to the neuron-specific chromatin remodeling complex (nBAF complex). Nevertheless, the relationship of this gene with IL-10 needs to be elucidated.

#### 3.3.3. Macrophage Inflammatory Protein 1α

Another association was rs75237116 at 10p13 with MIP-1α (CCL3) (*p* = 2.28 × 10^−9^). The SNP is located in *CAMK1D* and in proximity of *MIR4480*, *MIR548Q*, and *RNU6ATAC39P*. *CAMK1D* is involved in regulation of granulocyte function, activation of *CREB* (cAMP response element binding protein)-dependent gene transcription, aldosterone synthesis, differentiation and activation of neutrophil cells, and apoptosis of erythroleukemia cells [30]. The gene was reported to be associated with coronary artery aneurysm within Kawasaki disease [34]. Therefore, we consider *CAMK1D* as the plausible candidate here.

Further associations were found at 7q11.23 (rs145122044, *p* = 1.55 × 10^−8^), 11q25 (rs11223001, *p* = 1.73 × 10^−8^), 15q14 (rs118008913, *p* = 4.81 × 10^−8^), 17q22 (rs8082167, *p* = 1.22 × 10^−8^), and at Xq13.1 (rs3788792, *p* = 2.74 × 10^−9^). The lead SNPs on chromosomes 7, 11, and X are in the genes *UPK3B*, *NTM*, and *HDAC8*, respectively. However, biological relationships of these genes with the respective associated cytokines remain unclear.

### 3.4. Lookup of Cytokine-Coding Genes

We could identify significant associations for 40 SNPs in or nearby the corresponding gene from a total of 24,354 SNPs applying hierarchical false discovery rate control at 5%. Only two cytokines were involved in these associations, i.e. associations were found for only two candidate loci. Results can be found in Table S5. A total of 39 of these SNPs correspond to VEGF at locus 6p21.1. For 6p21.1, we found associations with multiple cancers [26] and large artery atherosclerotic stroke [27] in the literature. One significant SNP corresponds to MIP-1α (CCL3) on locus 17q12. The locus reveals associations with acute lymphoblastic leukemia [35], cervical cancer [36], and the fraction of exhaled nitric oxide values [37]. No associations were found for the coding genes of the other eight cytokines. 

## 4. Discussion

CAP is a disease affecting people of all ages with high mortality. Cytokines are of potential value to predict the future disease course but their context specific genetics is only partly understood. In this work, we performed a genome-wide association study and consecutive fine-mapping to elaborate genetic determinants of ten major cytokines measured in blood serum.

Among the ten cytokines there were five interleukins (1β, 6, 8, 10, and 12). Pro- and anti-inflammatory cytokines IL-6, IL-8, and IL-10 play an important role in the complex response of the human immune system due to CAP [38]. The influence of IL-12 on the expression and signaling pathways of VEGF and consequently on angiogenesis has already been demonstrated in tumour cells by [39] and for type 2 diabetes mellitus by [40]. An interaction of IL-12 with IL-10 could also be demonstrated. IL-12 activates the immune response of the T-helper cells type 1, characterised by the cytokine interferon-γ, and mediates the activation of macrophages for the elimination of the pathogen. This process is negatively regulated by IL-10 (T helper cell type 2). The ratio of IL-10/IL-12 can be used as a measure for the balance of pro- and anti-inflammatory mediators and thus can be used to assess the immune status [41]. However, this approach was not pursued in this work.

In our GWAS, we found genome-wide associations for five of the analysed ten cytokines, namely VEGF, IL-12, IL-1β, IL-10, and MIP-1α (CCL3). Genetic associations with cytokine VEGF at locus 6p21.1 have already been investigated in some studies. The two GWAS by [42] and [8] report a strong association of the variant rs6921438 (top SNP rs7763440; LD = 0.93) with VEGF concentration. The SNP is located 171 kb downstream of the *VEGFA* gene, which codes for VEGF. Thus, *VEGFA* is the plausible candidate gene. The known variant at this locus is also believed to influence the concentration of four other cytokines (IL-12, IL-7, IL-10, and IL-13) [8]. We could confirm these results by showing genome-wide significance of this locus with VEGF and IL-12 and nominal significance for IL-10. The cytokines IL-7 and IL-13 were not considered in our study.

For the cytokine IL-1β, the variants rs117439842 and rs9903904 at locus 17q21.32 were found in the present study. The latter association also confirms results of another GWAS [29]. The following candidate genes were discerned: *SCRN2*, *LRRC46*, and *SP2*. The *SP6* gene is associated with Hermansky–Pudlak syndrome (HPS), which is associated with pulmonary fibrosis. HPS is characterised by dysregulation of alveolar macrophages (AM) [43]. AM are known to secrete the cytokine IL-1β [44]. According to Gene Ontology annotation, *OSBPL7* is involved in the binding of cholesterol, while *MRPL10* plays a role in mitochondrial translation and the translation of viral mRNA. The latter is crucial for the spread of viruses in the organism. However, we would refrain from considering this gene as a candidate. Cholesterol plays an important role in pneumococcal disease [45]. Thus, *OSBPL7* is a plausible candidate gene here.

The gene *CAMK1D* appears in connection with two genome-wide significant SNPs at locus 10p13 for the cytokine MIP-1α (CCL3). The variant found, rs7902334, was already identified in 2016 by [34] in a GWAS on Kawasaki syndrome. *CAMK1D* is a protein-coding gene of the calcium/calmodulin-dependent protein kinases 1 family. As part of the CaMKK-CaMK1 signalling cascade, it regulates, among other things, the calcium-mediated granulocyte function and the activation of CREB-dependent transcription. In patients with CAP, a reduction in the level of CREB-regulated transcription is observed during recovery [46]. We therefore consider *CAMK1D* as the plausible candidate.

Several genes (*ARMC4*, *SP2*, *LRRC46*, *RSPH9*, and *ZMYND10*) assigned to associations are involved in primary ciliary dyskinesia (PCD), an autosomal recessive inherited disease. Mutations of a pool of approximately 250 genes can lead to structural and/or functional dysfunction of the motile cilia. The structure of these hair-like cellular extensions of epithelial cells in the respiratory tract follows a fixed pattern of microtubules (9 + 2) and associated structures including dynein arms and spokes. In the respiratory tract, motile cilia are responsible for the removal of mucus, thereby protecting it from infection and ensuring mucociliary clearance. Hjeij et al. have shown that the *ARMC4* gene plays an important role in anchoring the outer dynein arms. In the case of a defect, the affected cilia exhibit reduced beating frequencies and amplitudes or become immotile [47]. Some of the SNPs in the *ARMC4* gene region have high CADD scores, so that pathogenic effects of these variants can be assumed. The substitutions promote the development of defective proteins that disrupt the correct structure of the axoneme. For the cytokine VEGF, the study also revealed the cis-eQTL gene *RSPH9* at 6p21.1. This gene also codes for a component of the motile cilia, the so-called radial spoke head. The radial spokes support the order of the tubule pairs. Mutations of *RSPH9* can cause changes in the movement of motile cilia [48]. Of note, for a SNP at locus 9q34.12, another gene with a linkage to PCD was found. This is due to a trans-eQTL with the gene *ZMYND10*. Mutations of this gene can lead to the loss of the inner and outer dynein arm complexes and thus to immobility of the cilia [49]. Malfunctions in the coordinated movement of cilia can impede the removal of invading microorganisms from the respiratory tract and promote infections of the respiratory tract. Therefore, PCD patients often suffer from recurrent pneumonia. In summary, several associations point to genes involved in ciliary function providing a functional link towards liability to or severity of pneumonia, and with it, cytokine regulation.

The individual´s immune responses depends on various factors and are also affected by the pathogen which is known only for a small subset of patients of our cohort. The total sample size of this study was small, which is another limitation. Replication of our results in other studies are therefore required. Although our analyses were performed context-specific (baseline values at hospitalisation), cytokine response dynamics were not analysed.

## 5. Conclusions

This genome-wide association study revealed 14 loci, two of them already known, showing that cytokine response is genetically determined. Several functional genes assigned to loci are involved in primary ciliary dyskinesia making them biologically plausible. Larger sample sizes and time series are required to further corroborate and improve our findings. Functional studies should be initiated to test our candidate genes.

## Figures and Tables

**Table 1:** Overview of patient characteristics. Statistics are presented as median (minimum to maximum), respectively, absolute numbers (percentages). Total number of patients with cytokine measurement at baseline is *N* = 400.

Trait	Statistics
Age [years]	62 (18–94)
Males/Females	239 (60%)/161 (40%)
Body mass index [kg/m^2^]	26.3 (15.0–54.3)
Current smoking	117 (29%)
Years of smoking (current and former smoker)	14 (0–65)
Years of smoking (only current smoker)	25 (4–60)
Chronic kidney disease	39 (10%)
Chronic liver disease	9 (2%)
Diabetes	83 (21%)
Antibiotic therapy prior to hospitalisation	100 (25%)

**Table 2:** Results of genome-wide SNP association analyses. In the table we present all 14 loci with genome-wide significant associations (threshold 5 × 10^−8^). For each locus, lead SNP, corresponding cytoband and physical position (GRCh37), physically nearby genes (within ±250 kb), best associated trait, effect allele, other allele, effect allele frequency, and beta estimate of the additive model with standard error and *p*-value for the top associated cytokine are shown. We also present the size of the respective 95% and 99% credible sets. For loci 3p21.31, 11q25, and Xq13.1 the lead SNPs showed minor allele frequencies below 1% in the complete data set and, thus, were excluded from the reference data set for conditional and joint analysis and credible set analysis resulting in empty cells in the corresponding rows. Loci are presented in the order of their chromosomal position.

Locus	Lead SNP	Cytoband	Physical Position	Physical Nearby Genes (Distance to Lead SNP (kb))	Best Associated Trait	Effect Allele	Other Allele	Allele Frequency	Beta	SE	*p*-Value	#SNPs in Credible Set (First: 95%, Second: 99%)
#1	rs116606423	2p16.3	50123457	RPL7P13 (17), NRXN1 (22)	IL-1β	G	T	0.02	1.39	0.25	3.46 × 10^−8^	3484 3819
#2	rs139453626	3p21.31	47700006	SMARCC1 (0), CSPG5 (78), RN7SL870P (98), DHX30 (140)	IL-10	G	A	0.02	2.42	0.42	2.39 × 10^−8^	-
#3	rs7763440	6p21.1	43926708	C6orf223 (42), MRPL14 (150), TMEM63B (170), VEGFA (170)	VEGF	A	G	0.45	−0.65	0.07	1.58 × 10^−20^	10 12
#4	rs145122044	7q11.23	76485397	UPK3B (0), FDPSP7 (110), DTX2P1 (120), DTX2P1-UPK3BP1-PMS2P11 (120)	MIP-1α (CCL3)	C	T	0.01	1.54	0.27	1.55 × 10^−8^	3230 3631
#5	rs62505830	8p12	36121891	RN7SKP201 (2.1), MTND6P19 (15), RNU6-533P (45)	IL-1β	T	C	0.01	1.72	0.30	2.55 × 10^−8^	814 944
#6	rs36002018	9q34.12	133656053	ABL1 (0), EXOSC2 (76), PRDM12 (98), QRFP (110)	IL-10	T	A	0.02	1.75	0.31	4.52 × 10^−8^	2534 3160
#7	rs75237116	10p13	12655293	CAMK1D (0), MIR4480 (34), MIR548Q (110), RNU6ATAC39P (160)	MIP-1α (CCL3)	T	C	0.01	1.64	0.27	2.28 × 10^−9^	3945 4541
#8	rs6481492	10p12.1	28207393	ARMC4 (0), RPL36AP55 (13), MPP7 (130), RN7SKP132 (130)	IL-1β	C	T	0.06	0.79	0.13	2.76 × 10^−9^	29 50
#9	rs11223001	11q25	132161625	NTM (0), NTM-IT (6.6), OPCML (120), RNU6-1182P (230)	MIP-1α (CCL3)	G	A	0.01	1.56	0.27	1.73 × 10^−8^	-
#10	rs118008913	15q14	39036672	C15orf53 (44), RASGRP1 (180)	MIP-1α (CCL3)	G	A	0.02	1.10	0.20	4.81 × 10^−8^	3397 3761
#11	rs117439842	17q21.32	45933872	SP6 (0.63), SCRN2 (15), LRRC46 (19), MRPL10 (25), OSBPL7 (35), SP2 (40)	IL-1β	T	G	0.01	1.96	0.32	1.51 × 10^−9^	10 23
#12	rs8082167	17q22	52494156	ISCA1P3 (61)	MIP-1α (CCL3)	T	C	0.02	1.31	0.23	1.22 × 10^−8^	2838 3247
#13	rs76920584	18q21.2	51386971	-	IL-1β	C	T	0.01	1.49	0.26	2.11 × 10^−8^	194 1687
#14	rs3788792	Xq13.1	71585878	HDAC8 (0), RNU2-68P (11), CITED1 (59), PIN4 (63)	MIP-1α (CCL3)	T	C	0.02	1.17	0.19	2.74 × 10^−9^	-

## Data Availability

Data Availability Statement: Patient agreements of the PROGRESS study do not cover public availability of the data. Data requests can be addressed to the PROGRESS/CAPNETZ consortium (https://www.capnetz.de/html/progress/project).

## Supplementary Materials

The following are available online at https://www.mdpi.com/article/10.3390/genes13010111/s1, Figure S1: Regional Association Plots, Table S1: Sample Sizes for Combined Cytokine and Genetical Data, Table S2: Genomic Inflation Factors, Table S3: Results of Genome-Wide Association Study, Table S4: Results of MetaXcan Analysis, Table S5: Lookup Coding Genes.
