# Supplementary figures and images for: Genetic Regulation of Cytokine Response in Patients with Acute Community-Acquired Pneumonia
Source: Genes (Basel). 2022 Jan 6;13(1):111. doi: 10.3390/genes13010111 (PMC8774373; doi:10.3390/genes13010111)

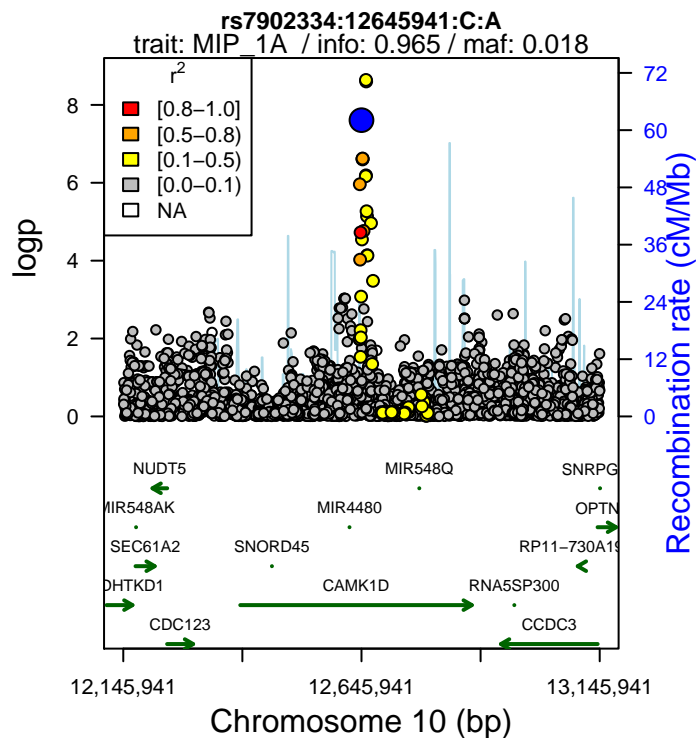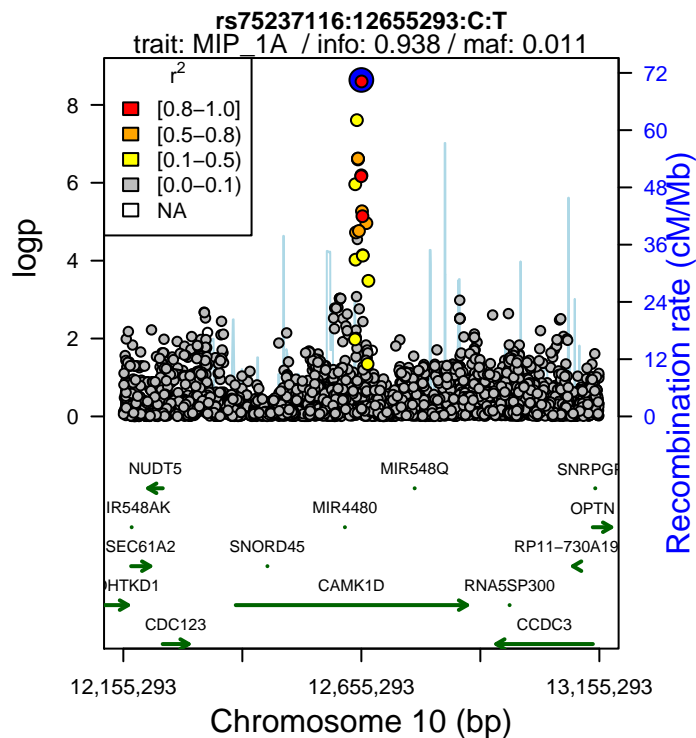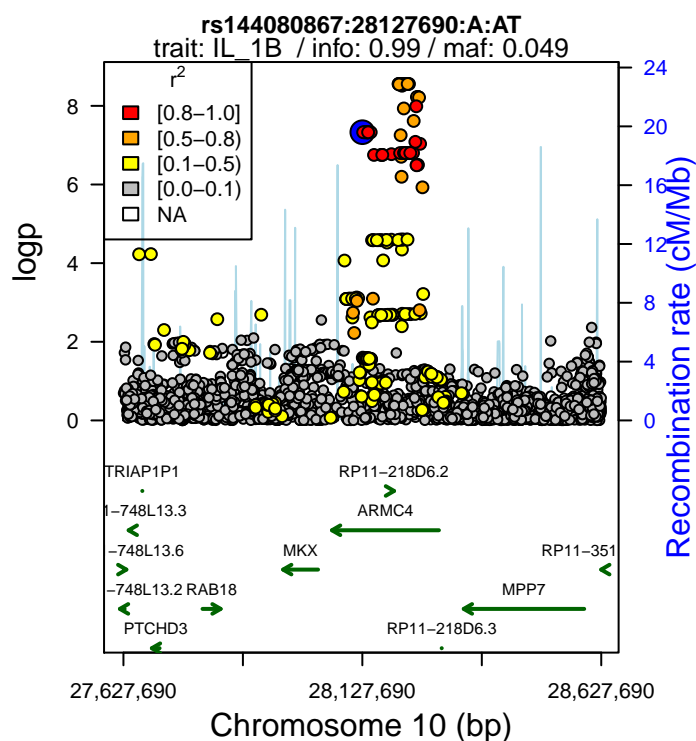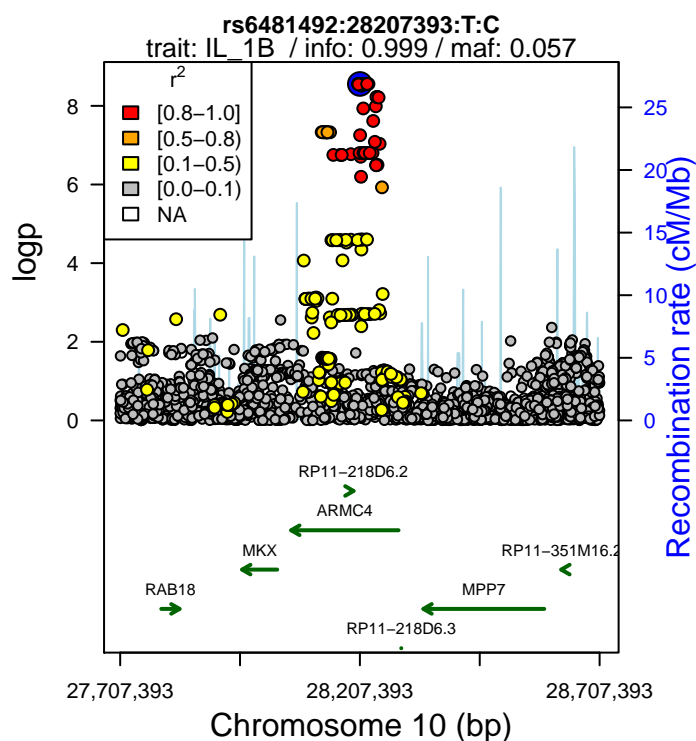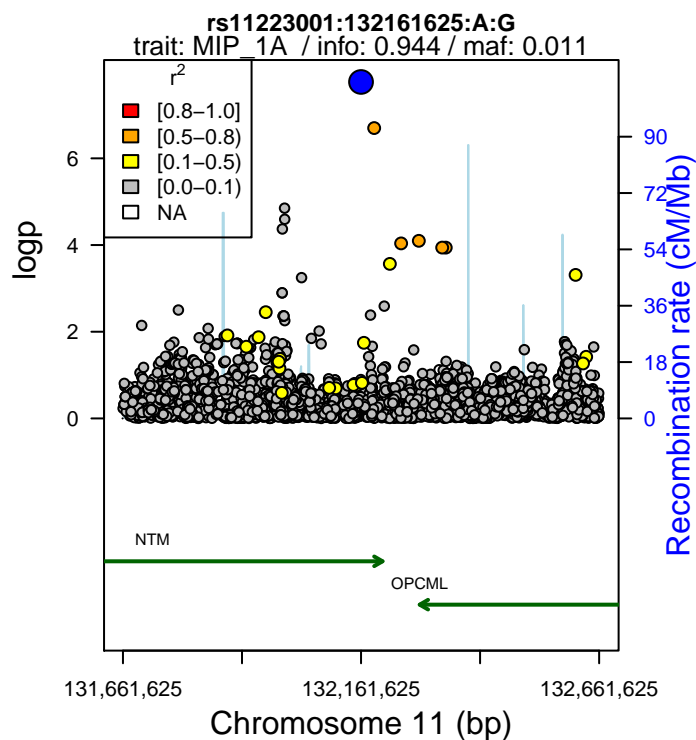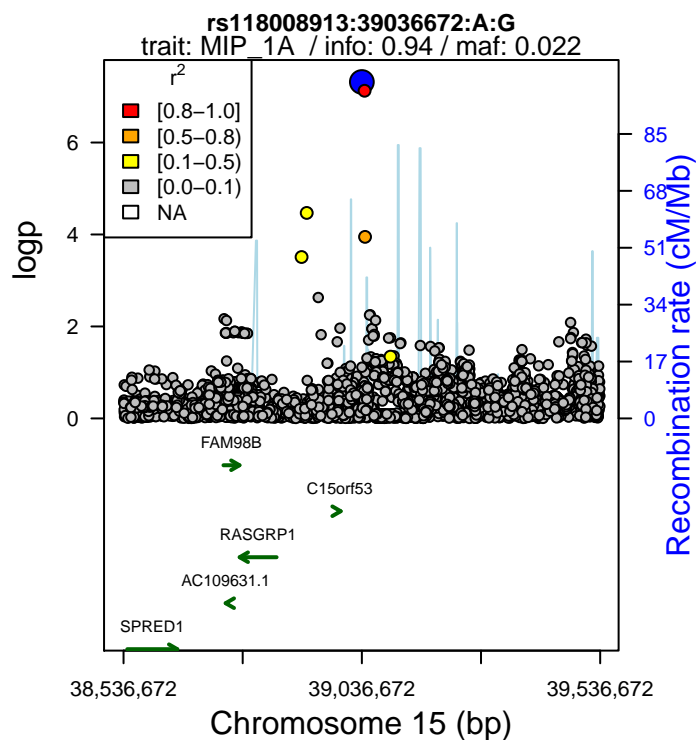

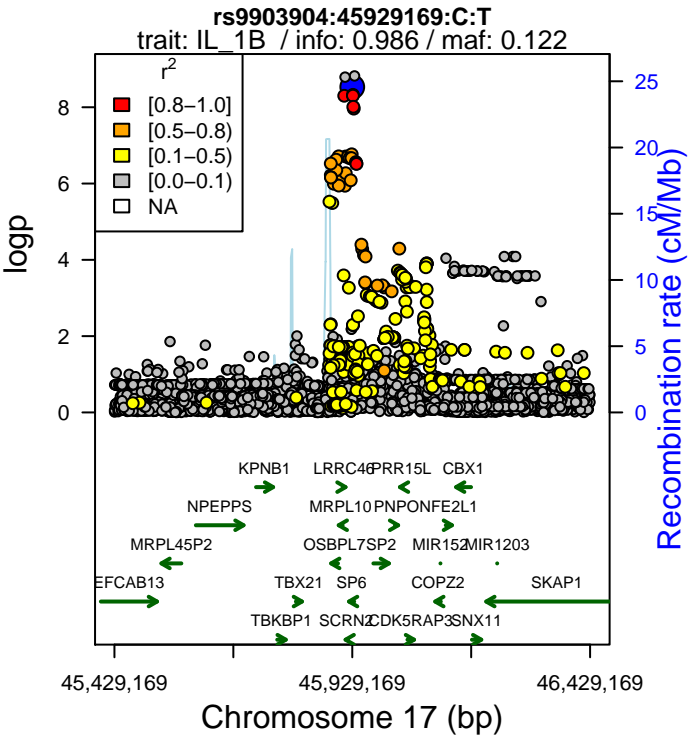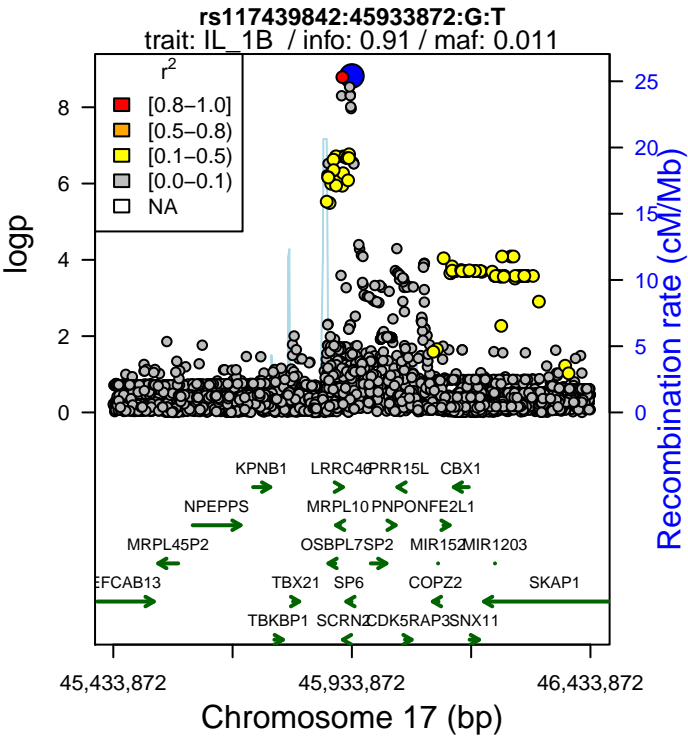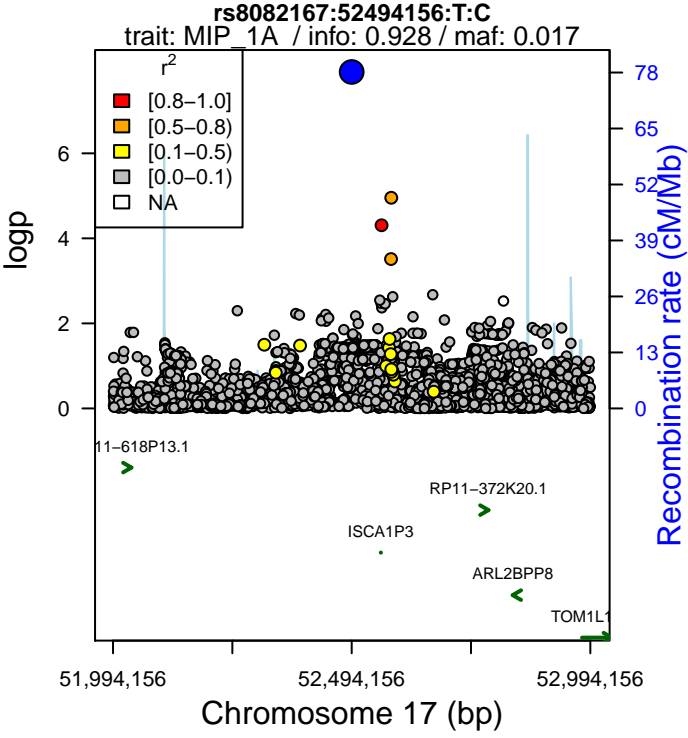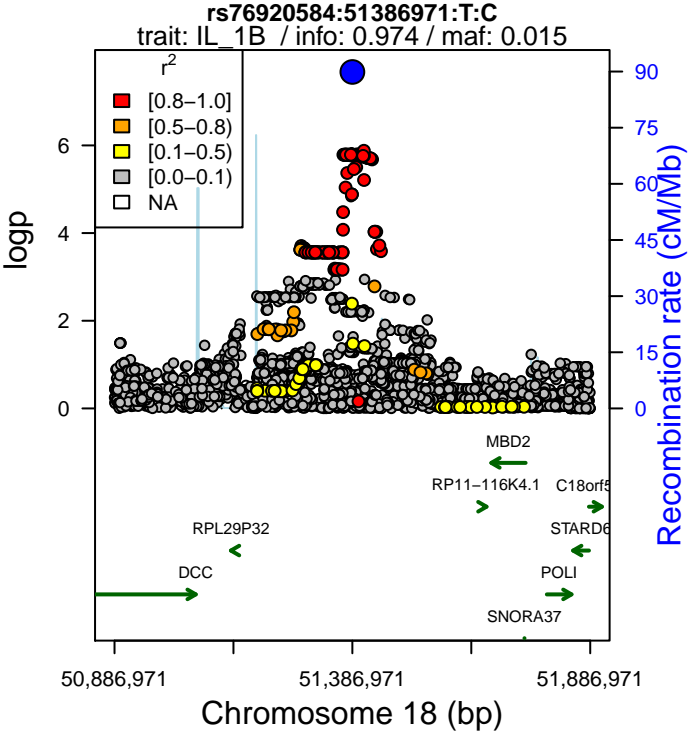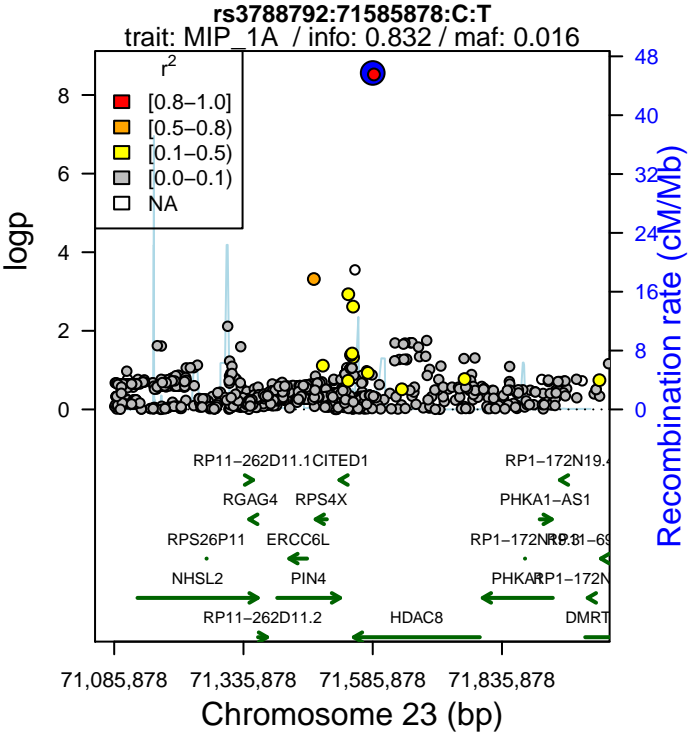

Supplement: Supplementary file 1 [file genes-13-00111-s001.zip › supplementary_figure_s1_20211127.pdf]
